# Practitioners and the Lay Press

**Published:** 1905-07-01

**Authors:** 


### Practitioners and the Lay Press.

Amidst the various questions which at occasional intervals stir and agitate the mind of the profession that of the appearance of individual practitioners in the columns of the lay press is not the least piquant. Even those press notices which arise from social or accidental circumstances receive a more or less suspicious scrutiny and are apt to call forth suggestive remarks about Mr. Vincent Crummles and the "way in which these things get into the papers." Whilst, speaking generally, open discussion of professional subjects in papers and journals intended for the general public obtains not unnaturally the most sinister interpretation. All this, it must be allowed, is on the whole a fair and reasonable attitude, seeing that the unwritten law of the profession is opposed to actions on the part of its individual members which tend to attract the attention of the general public, and to suggest the possession of superior knowledge and skill. A precise illustration of this principle may be found in the recent attempt of the most representative organisation of the profession to persuade editors of daily and other papers to abstain from mentioning the names of practitioners in connection, for example, with the illnesses of public personages, or otherwise. In all this there is of course a logical and self-consistent policy. Individuals may differ from the wisdom of the policy, but its coherence is beyond question. On the other hand, it is competent to contend that the wise policy is directly the reverse of the one which commands the general adhesion of professional opinion. It is open to argument that medical practitioners, like those engaged in commercial pursuits, should be free to adopt, each for himself, whatever method he may prefer for the purpose of obtaining the attention and confidence of a section of the suffering public. His plans, it might be urged, may be left to his own taste and discretion and sense of self-respect. In so far as these standards are low and peculiar the individuals concerned may be expected to suffer in professional and public repute. Here again is a definite and self-consistent policy. It is not accepted by the profession in this country, but it has not altogether lacked sponsors, and it must at least be recognised as suggesting a principle capable of affording a practical guidance in the affairs of life and conduct. But, for good or ill, the voice of the profession disclaims the mode of the advertiser, and in theory at least offers its subscription to the view that public and professional interests are not promoted by the expression of opinions on medical topics by members of the profession in the columns of the lay press. Now it is manifest if this rule is to be enforced it must apply with impartial incidence to all practitioners, whether distinguished or otherwise. Exceptions and limitations in such a matter are out of the question. It will not do to condemn as "rank blasphemy" in the private that which in the captain is condoned as a mere "choleric word." There must be one weight and one measure. For it is inevitable that unless this rigidity of application is demanded the principle and the practice it inculcates will both soon cease to exist. The earlier and slighter departures from the rule will be quoted to justify later and larger breaches. Moreover, such quotation cannot be impugned, as the rule either exists or it does not exist, and if the former it is as definitely defied by one effort towards publicity as by another. Hence those who believe—and we count ourselves among the number—that the welfare of patients and practitioners alike is promoted by the absence of professional proclamations in the public press are particularly bound to take note of early and slight departures from the established custom, for in these the abolition of the custom is in essence contained. It is for this reason that we express regret in seeing a tendency on the part of some members of the profession to grant interviews on medical topics to representatives of the daily press. Within the last few weeks, at least two such interviews have appeared in the cheaper London daily papers, and we think the practice should at once receive professional condemnation. That the practitioners concerned occupy the highest rank in the medical hierarchy makes the position all the more serious. If this method is accessible to West End consultants, it must be open also to others. Either the stealing of horses must be universally forbidden, or no one can be condemned for looking over the hedge. The profession has elected on the question of publicity one out of the only two possible policies. Unless this policy is universally and impartially applied it will necessarily disappear and be replaced by its rival.

